# Opportunistic Infections in COVID-19: A Systematic Review and Meta-Analysis

**DOI:** 10.7759/cureus.23687

**Published:** 2022-03-31

**Authors:** Nithin Kurra, Priyanka Isaac Woodard, Nikhila Gandrakota, Heli Gandhi, Srinivasa Rao Polisetty, Song Peng Ang, Kinjalben P Patel, Vishwaj Chitimalla, Mirza M Ali Baig, Gayathri Samudrala

**Affiliations:** 1 Department of Neurology, University of Nebraska Medical Center, Omaha, USA; 2 Medicine and Surgery, Volgograd State Medical University, Volgograd, RUS; 3 Department of Family Medicine, Emory University, Atlanta, USA; 4 Medicine and Surgery, Manipal Academy of Higher Education, Manipal, IND; 5 Medicine and Surgery, International Medical University, Kuala Lumpur, MYS; 6 Medicine and Surgery, Smt. B. K. Shah Medical Institute & Research Centre, Vadodara, IND; 7 Medicine and Surgery, Shri B M Patil Medical College Hospital and Research Centre, Bijapur Lingayat District Educational (BLDE) University, Vijayapura, IND; 8 Department of Anaesthesiology, Dow University of Health Sciences, Karachi, PAK; 9 Obstetrics and Gynecology, National Board of Examinations, New Delhi, IND; 10 Medicine and Surgery, Dr. N. T. Ramarao University of Health Sciences, Vijayawada, IND

**Keywords:** covid-19, bacterial coinfection in covid-19, sars-cov-2, coronavirus-associated pulmonary aspergillosis, respiratory coinfections, superinfections, opportunistic fungal infection, covid-19 co-infection, secondary infections, opportunistic infections

## Abstract

The prevalence, incidence, and characteristics of bacterial infections in patients infected with severe acute respiratory syndrome coronavirus 2 are not well understood and have been raised as an important knowledge gap. Therefore, our study focused on the most common opportunistic infections/secondary infections/superinfections in coronavirus disease 2019 (COVID-19) patients.

This systematic review and meta-analysis was conducted according to the Preferred Reporting Items for Systematic Reviews and Meta-Analyses. Eligible studies were identified using PubMed/Medline since inception to June 25, 2021. Studies meeting the inclusion criteria were selected. Statistical analysis was conducted in Review Manager 5.4.1. A random-effect model was used when heterogeneity was seen to pool the studies, and the result was reported as inverse variance and the corresponding 95% confidence interval.

We screened 701 articles comprising 22 cohort studies which were included for analysis. The pooled prevalence of opportunistic infections/secondary infections/superinfections was 16% in COVID-19 patients. The highest prevalence of secondary infections was observed among viruses at 33%, followed by bacteria at 16%, fungi at 6%, and 25% among the miscellaneous group/wrong outcome.

Opportunistic infections are more prevalent in critically ill patients. The isolated pathogens included Epstein-Barr virus, *Pseudomonas aeruginosa*, *Escherichia coli*, *Acinetobacter baumannii*, *Hemophilus influenza*, and invasive pulmonary aspergillosis. Large-scale studies are required to better identify opportunistic/secondary/superinfections in COVID-19 patients.

## Introduction and background

The coronavirus disease 2019 (COVID-19) pandemic has been associated with fatal outcomes. Recent studies have shown that the primary route of transmission of severe acute respiratory syndrome coronavirus 2 (SARS-CoV-2) is through respiratory droplets [1,2]. Studies have also shown that 25% of older individuals affected with influenza acquire secondary bacterial infections [3,4]. Moreover, these individuals have been reported to have superinfections and coinfections with SARS-CoV-2 [5-7]. However, there is limited data on the frequency of viral, bacterial, or fungal coinfections and superinfections in COVID-19 patients [5-7].

Numerous opportunistic infections have been reported in COVID-19 patients, including *Aspergillus* spp., *Candida* spp., *Cryptococcus neoformans*, *Pneumocystis jirovecii* (*carinii*), mucormycosis, cytomegalovirus (CMV), herpes simplex virus (HSV), *Strongyloides stercoralis*, *Mycobacterium tuberculosis*, and *Toxoplasma gondii* infections [8]. A recent meta-analysis reported coinfections and superinfections in 19% and 24% of COVID-19 patients, respectively, both being associated with the risk of increased mortality [9].

The prevalence, incidence, and characteristics of bacterial infections in patients infected with SARS-CoV-2 are not well understood and have been raised as a significant knowledge gap. Therefore, we conducted a systematic review and meta-analysis on opportunistic infections, secondary infections, and superinfections in COVID-19 patients.

## Review

Methodology

Data Sources and Search Strategy

This systematic review and meta-analysis were performed according to the Preferred Reporting Items for Systematic Reviews and Meta-Analyses (PRISMA) guidelines [10]. We searched data from PubMed/Medline from their inception to June 25, 2021, using the following keywords: “bacterial infections AND covid,” “coinfections AND covid,” “fungal infections AND covid,” “opportunistic infections AND covid,” “opportunistic pulmonary AND covid,” “secondary infections AND covid,” “superinfections AND covid.” We also screened review articles, cohort studies, randomized controlled trials (RCTs), and meta-analyses for further relevance.

Study Selection

Our eligibility criteria for the studies, abbreviated as PECOS, included the following: (1) P (population): COVID-19 patients; (2) E (exposure): superinfection; (3) C (control): none; (4) O (outcome): pooled prevalence of superinfection in COVID-19; (5) S (studies): human-based RCTs and cohort studies published in English only.

Statistical Analysis

Statistical analysis was done using the Review Manager (version 5.4.1; The Nordic Cochrane Centre, Copenhagen) and the Cochrane Collaboration tool. We pooled the data from studies using a random-effects model when heterogeneity was present. We analyzed the results by calculating the inverse variance (IV) with respective 95% confidence intervals (CIs). Any differences between the subgroups were determined using the chi-square test. We performed a sensitivity analysis to look for any single study that could be driving the results and to assess the cause of high heterogeneity.

I^2^ is the degree of inconsistency measured (range: 0-100%) across studies in a meta-analysis. It quantifies the effect of heterogeneity rather than chance. Heterogeneity scales were considered as follows based on the Cochrane handbook: I^2^ = 25-60%, moderate; 50-90%, substantial; 75-100%, considerable heterogeneity. P-values of <0.1 indicated significant heterogeneity [11]. P-values of <0.05 were considered significant for all analyses.

Prevalence was calculated using raw data. This along with other extracted information was used to find standard errors using the formula:

$SE = \sqrt{\frac{p\times \left ( 1-p \right )}{n}}$

Where p is the prevalence and n is the number of COVID-19 patients. The prevalence and standard error of each study were then entered into the Review Manager through the IV method to compute pooled prevalence along with 95% CIs.

Data Extraction and Quality Assessment of Studies

We searched the electronic databases, exported the studies to the EndNote Reference Library software, version 20.0.1 (Clarivate Analytics), and removed any duplicates after screening. We extracted the data and assessed the quality of the cohort studies using the Newcastle-Ottawa Scale (NOS) where a score of 1-5 was considered high risk for bias, 6-7 as moderate, and >7 as low (Table 1).

**Table 1:** Quality assessment of cohorts using the New Ottawa Scale.

Studies	Selection (Maximum 4)				Comparability (Maximum 2)	Outcome (Maximum 3)			Total score
	Representativeness of the exposed cohort	Selection of the non-exposed cohort	Ascertainment of exposure	Demonstration that outcome of interest was not present at the start of the study	Comparability of cohorts on the basis of the design or analysis	Assessment of outcome	Was follow-up long enough for outcomes to occur	Adequacy of follow-up of cohorts	
Sharifipour, et al. 2020 [12]	1	1	1	0	1	1	1	1	7
Sharov 2020 [13]	1	1	1	0	1	1	1	1	7
Asmarawati, et al. 2021 [14]	1	1	1	1	1	1	1	1	8
Garcia-Vidal, et al. 2021 [15]	1	1	1	1	1	1	1	1	8
Ripa, et al. 2021 [16]	1	1	1	1	1	1	1	1	8
Russell, et al. 2021 [17]	1	1	1	1	1	1	1	1	8
Søgaard, et al. 2021 [18]	1	1	1	1	1	1	1	1	8
Razazi, et al. 2020 [19]	1	1	1	0	1	1	1	1	7
White, et al. 2020 [20]	1	1	1	1	1	1	1	1	8
Li, et al. 2020 [21]	1	1	1	1	1	1	1	1	8
Bayram, et al. 2021 [22]	1	1	1	1	1	1	1	1	8
Lahmer, et al. 2021 [23]	1	1	1	1	1	1	1	1	8
Pinatdo, et al. 2021 [24]	1	1	1	0	1	1	1	1	7
Segrelles-Calvo, et al. 2021 [25]	1	1	1	1	1	1	1	1	8
Gouzien, et al. 2021 [26]	1	1	1	1	1	1	1	1	8
Paolucci, et al. 2020 [27]	1	1	1	1	1	1	1	1	8
Zhang, et al. 2020 [28]	1	1	1	1	1	1	1	1	8
Bardi, et al. 2021 [29]	1	1	1	1	1	1	1	1	8
Falcone, et al. 2021 [30]	1	1	1	1	1	1	1	1	8
Khurana, et al. 2021 [31]	1	1	1	1	1	1	1	1	8
Kubin, et al. 2021 [32]	1	1	1	1	1	1	1	1	8
Kumar, et al. 2021 [33]	1	1	1	1	1	1	1	1	8

Results

Literature Search Results

**Figure 1:**
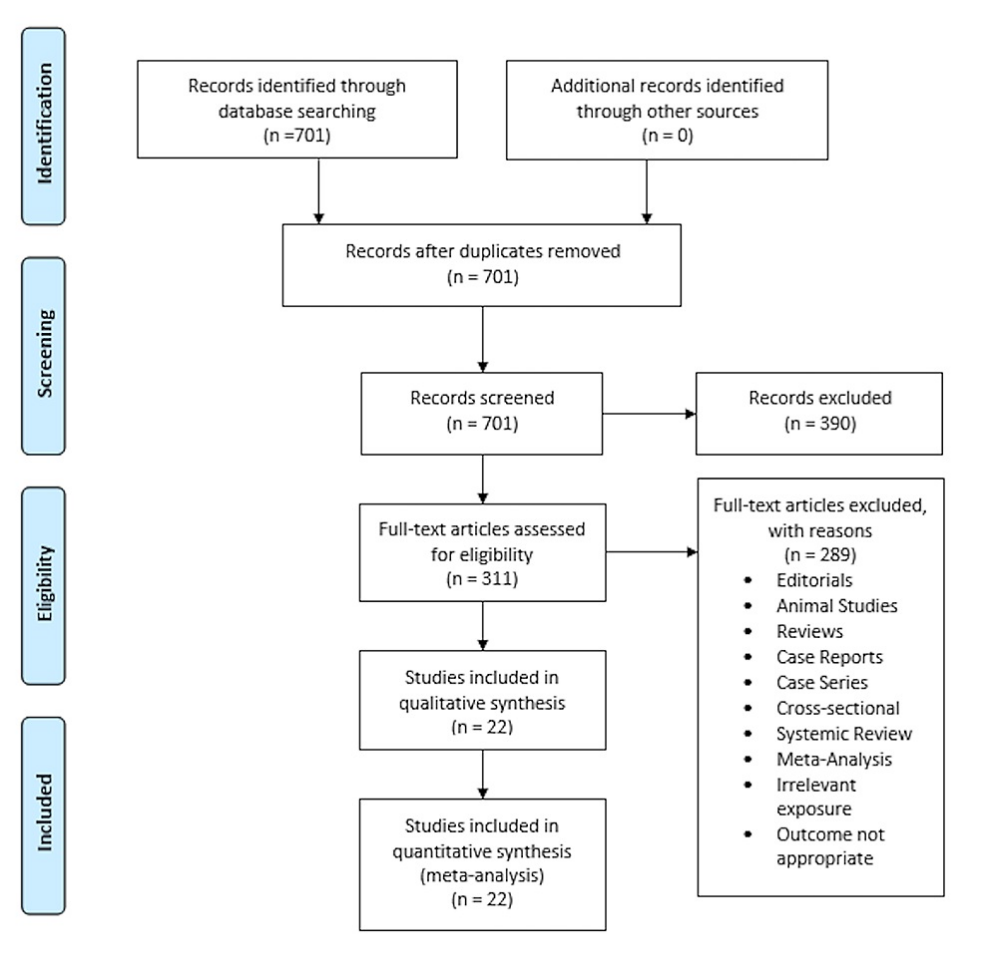
PRISMA flowchart. PRISMA: Preferred Reporting Items for Systematic Reviews and Meta-Analyses

Approximately 701 studies were searched initially from the electronic databases. There were no duplicates. We excluded 30 articles based on their title and abstracts. The full texts from 311 studies were examined to be included after excluding studies based on titles and abstracts. Finally, 22 studies were included in the quantitative analysis. Figure 1 shows our literature search results.

Study Characteristics

Table 2 provides the basic characteristics of included studies. In total, 22 studies included a total of 13,550 patients [12-33]. Overall, 13 studies were from Europe [15-20,22,23,25-27,29,30], five were from Asia [12,14,21,28,31], two were from the United States [32,33], one was from Russia [13], and one was from Mexico [24]. Two studies were part of the bacteria, virus, and fungi group [15,18]; one was part of bacteria and fungi only [16]; four were part of the bacteria-only group [12-14,17]; eight were part of the fungi-only group [19-26]; one was in the virus-only group [27], and six were in the miscellaneous group [28-33].

**Table 2:** Basic characteristics of the selected studies. ICU: intensive care unit; C-ARDS: COVID-19-associated ARDS; NC-ARDS: non-SARS-CoV-2 viral ARDS; ARDS: acute respiratory distress syndrome; VAP: ventilator-associated pneumonia; EBV: Epstein-Barr virus

Author	Country	Study type	Sample size	Patients died (%)	Subgroup	Infections
Sharifipour, et al. 2020 [12]	Iran	Cohort	19	95%	Bacteria	90% of bacterial coinfections were due to *Acinetobacter baumannii*, and 10% of bacterial coinfections were due to *Staphylococcus aureus*
Sharov 2020 [13]	Russia	Cohort	Set 1: 3,382	Set 1: 18.5%	Bacteria	Set 1: 41.5% of secondary bacterial infections were due to *Streptococcus pneumoniae*, *Staphylococcus aureus*, and *Hemophilus influenzae*
			Set 2: 1,204	Set 2: 7.39%	Bacteria	Set 2: 35.96% were secondary pneumonia and coinfections
Asmarawati, et al. 2021 [14]	Indonesia	Cohort	218	16.28%	Bacteria	23% were bacterial coinfections and 77% were secondary bacterial infections. The most common bacteria were *Acinetobacter baumannii*, followed by *Klebsiella pneumoniae*, *Pseudomonas aeruginosa*, *Escherichia coli*, *Enterobacter cloacae* complex, and *Staphylococcus haemolyticus*
Garcia-Vidal, et al. 2021 [15]	Spain	Cohort	989	9.8%	Bacteria, fungi, and virus	2.5% bacterial coinfections were due to *Streptococcus pneumoniae* and *Staphylococcus aureus*. 3.8% bacterial superinfections were due to *Pseudomonas aeruginosa* and *Escherichia coli*. 0.7% were hospital-acquired fungal superinfections caused by *Aspergillus fumigatus* and *Candida albicans*
Ripa, et al. 2021 [16]	Italy	Cohort	731	26.5%	Bacteria and fungi	9.3% were secondary infections, the majority caused by bloodstream infections. 69.7% were due to coagulase-negative staphylococci, 30.4% were due to *Acinetobacter baumannii*, and 21.7% are due to *Escherichia coli*. Secondary infection was frequently seen in patients admitted to the ICU in two (45/86) days compared to patients never admitted to ICU or admitted to ICU after two days
Russell, et al. 2021 [17]	UK	Cohort	1,107	31.5%	Bacteria	70.6% of the secondary infections were due to *Staphylococcus aureus* and *Hemophilus influenzae*
Søgaard, et al. 2021 [18]	Switzerland	Cohort	162	10.5%	Bacteria, fungi, and virus	36.6% were hospital-acquired secondary bacterial infections. The most common cause is Enterobacteriaceae. 1.7% were hospital-acquired fungal infections caused by *Aspergillus fumigatus* and *Candida albicans*
Razazi, et al. 2020 [19]	France	Cohort	C-ARDS: 90	C-ARDS: 41%	Fungi	16% were bacterial coinfections in C-ARDS versus 48% in NC-ARDS. The most common organisms associated with VAP were Enterobacteriaceae. There were no proven aspergillosis cases in the entire study. Probable aspergillosis and putative aspergillosis were less common in C-ARDS than in NC-ARDS patients
			NC-ARDS: 82	NC-ARDS: 33%		
White, et al. 2020 [20]	UK	Cohort	135	38%	Fungi	14.1% were due to aspergillosis
Li, et al. 2020 [21]	China	Cohort	1495	49%	Fungi	6.8% were secondary bacterial infections. 35.8% were due to *Acinetobacter baumannii*, 30.8% were due to *Klebsiella pneumoniae*, and 6.3% were due to *Streptococcus maltophilia*
Bayram, et al. 2021 [22]	Turkey	Cohort	11	63.6%	Fungi	Mucormycosis was seen in all patients. Of these, 63.6% had orbital apex syndrome, 36.4% had orbital cellulitis, and 54.5% had endophthalmitis
Lahmer, et al. 2021 [23]	Germany	Cohort	32	19%	Fungi	34% were COVID-19-associated invasive pulmonary aspergillosis
Pintado, et al. 2021 [24]	Mexico	Cohort	83	31%	Fungi	19.3% were COVID-19-associated invasive pulmonary aspergillosis
Segrelles-Calvo, et al. 2021 [25]	Spain	Cohort	215	86%	Fungi	22.8% had opportunistic invasive fungal infections and 5.4% had aspergillosis.
Gouzien, et al. 2021 [26]	France	Cohort	53	37.7%	Fungi	1.9% were COVID-19-associated invasive pulmonary aspergillosis
Paolucci, et al. 2021 [27]	Italy	Cohort	104	None	Virus	95.2% of the ICU patients and 83.6% of the subintensive care unit patients had EBV DNA infection
Zhang, et al. 2020 [28]	China	Cohort	612	36.36%	Miscellaneous	57.89% were secondary infections. Of these, 50% were due to Gram-negative bacteria, 26.92% were due to Gram-positive bacteria, 11.54% were due to virus, 7.69% were due to fungi and 3.85% were due to others. The most common pathogens were *Klebsiella pneumoniae*, *Enterococcus faecium*, *Acinetobacter baumannii*, HSV1
Bardi, et al. 2021 [29]	Spain	Cohort	140	36%	Miscellaneous	The most frequent bacteria with primary BSI were *Enterococcus faecium* (43%), followed by *Enterococcus faecalis* (21%), and coagulase-negative staphylococci (CNS) (11%). Gram-positive bacteria were the most common cause of CRBSI (CNS 54%, *E. faecium* (17%), *E. faecalis* (8%)), 17% of CRBSI infections were caused by *Candida albicans*. *Pseudomonas aeruginosa* is the primary pathogen seen in patients with VAP (38%) and tracheobronchitis (33%). *Aspergillus* spp. were isolated in three cases of LRTI. *E. faecium* (44%) and *E. faecalis* (28%) were the most common causes of UTI
Falcone, et al. 2021 [30]	Italy	Cohort	315	18.8%	Miscellaneous	21.9% were superinfections. 44.9% were caused by enterobacterales. *Klebsiella pneumoniae* was the most common cause. 15.6% were caused by non-fermenting Gram-negative bacilli. *Pseudomonas aeruginosa* was the most common cause. 15.6% were caused by Gram-positive bacteria. *Enterococcus* was the most common cause. 5.5% are caused by fungi. *Candida albicans* was the most common cause
Khurana, et al. 2021 [31]	India	Cohort	290	33%	Miscellaneous	13% were secondary infections. The most common pathogen was *K. pneumoniae* (33%), followed by *Acinetobacter baumannii* (32%)
Kubin, et al. 2021 [32]	USA	Cohort	516	21%	Miscellaneous	6% were community-associated coinfections. The most common organisms were *Escherichia coli *(31%), *S. aureus* (11%), *Proteus mirabilis* (8%), and *Klebsiella pneumoniae* (8%). 12% were healthcare-associated infections, of which 57% were caused by Gram-negative bacteria. 19% are fungal infections. *Candida* was the most common infection. 17% of infections were caused by *Candida*
Kumar, et al. 2021 [33]	USA	Cohort	1,565	40.7%	Miscellaneous	3.7% were healthcare-associated infections. Of these, 31.5% were due to Gram-positive infections. The most common causes were *Staphylococcus aureus* and *Enterococcus*. 53.4% were due to Gram-negative infections. The most common causes were *Pseudomonas*, *E. coli*, and *Klebsiella*. 15% were fungal infections. *Candida *was the most common cause

Common bacteria present in the studies were *Acinetobacter baumannii*, *Staphylococcus aureus*, *Escherichia coli*, *Pseudomonas aeruginosa*, and *Hemophilus influenza*. Common fungi included *Aspergillus* and *Candida* species. Few virus species were isolated that might have caused infections such as Epstein-Barr virus (EBV). Table 2 highlights the prevalence of death within these studies. Most deaths were reported by Sharifipour et al., while the majority of other studies with a larger population size had a mortality rate between 10% and 40% [12].

Publication Bias and Quality Assessment

**Figure 2:**
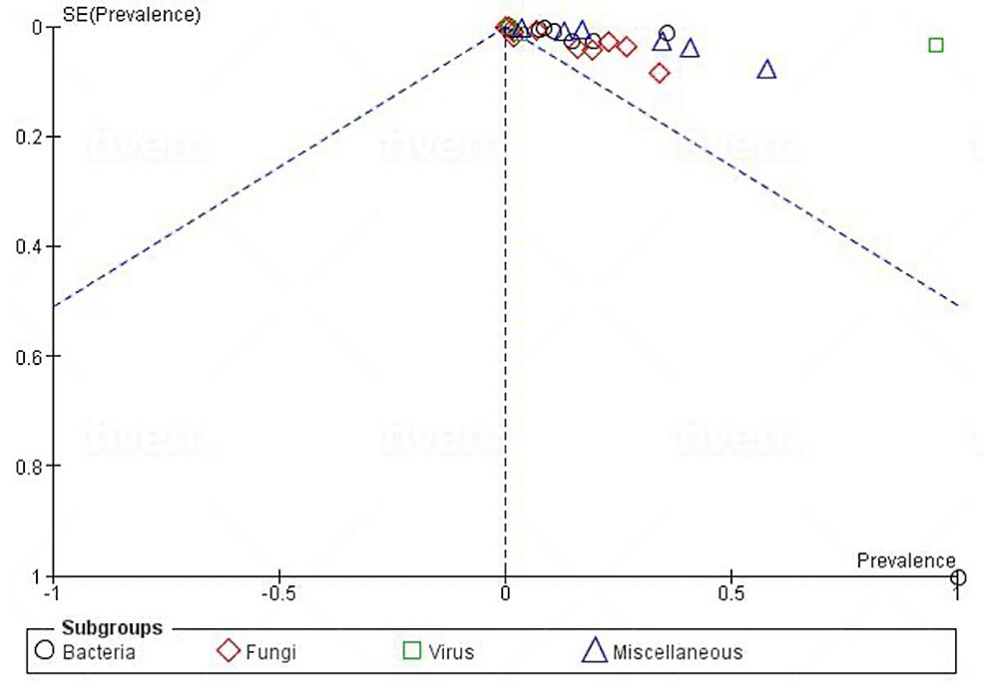
Funnel plot showing publication bias.

A funnel plot showing asymmetry which suggests publication bias is shown in Figure 2. Overall, three studies had a moderate risk of bias [12,19,24] while 19 studies had a low risk of bias [13-18,20-23,25-33].

Results of the Meta-Analysis

**Figure 3:**
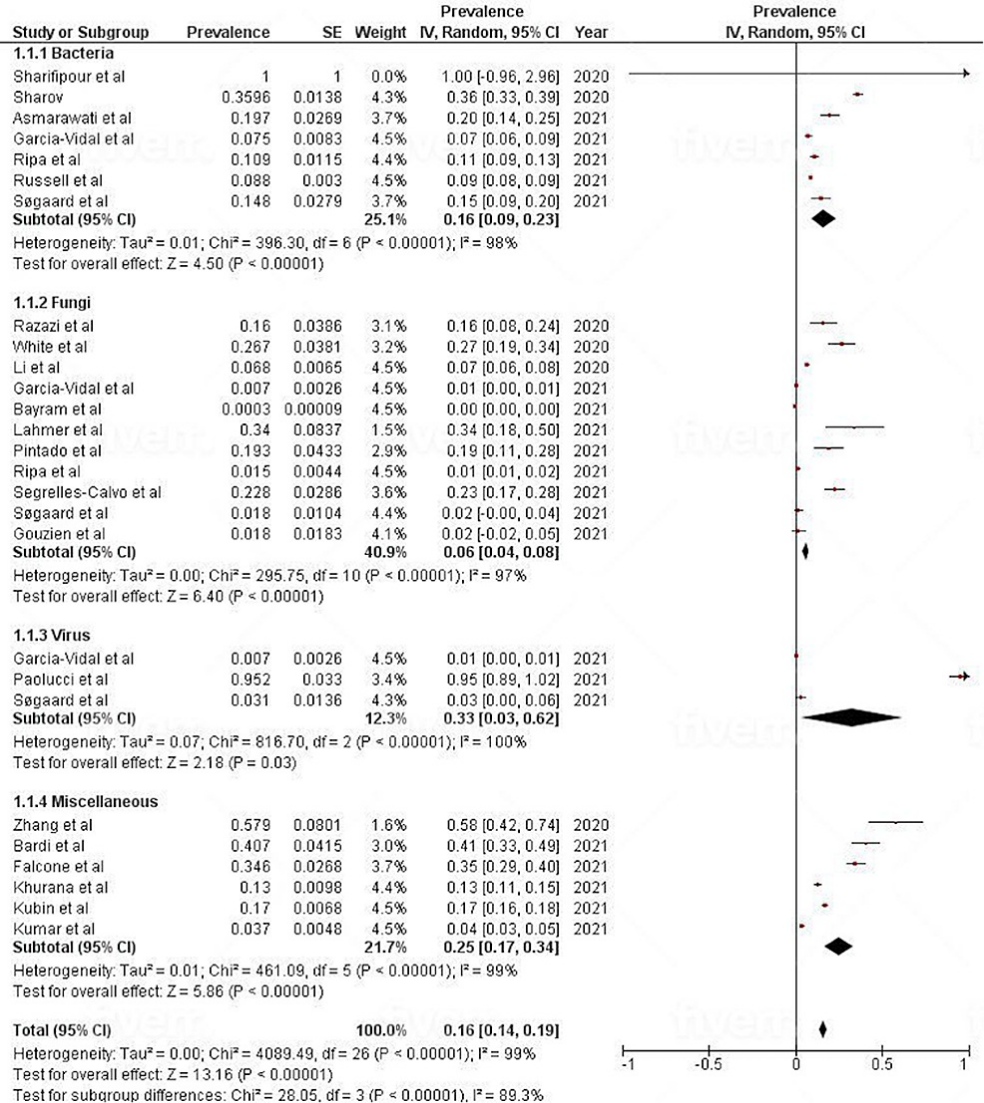
Forest plot showing pooled prevalence of superinfections in COVID-19 patients. COVID-19: coronavirus disease 2019

The pooled prevalence of opportunistic infections/secondary infections/superinfections in COVID-19 patients was 16% (95% CI = 14-19%; I^2^ = 99%) (Figure 3). Our study showed the highest prevalence of secondary infections among viruses at 33% (95% CI = 3-62%), while it was 16% (95% CI = 9-23%) among the bacteria subgroup, 6% (95% CI = 4-8%) among the fungi subgroup, and 25% (95% CI = 17-34%) among the miscellaneous group/wrong outcome. Most studies showed significant results, but few studies showed non-significant results. Sharifipour et al. [12] in the bacteria subgroup; Garcia-Vidal, et al. [15], Bayram et al. [22], Søgaard et al. [18], and Gouzien et al. [26] in the fungi subgroup; and Garcia-Vidal et al. [15] and Søgaard et al. [18] in the virus subgroup showed non-significant prevalence. All studies in the miscellaneous subgroup were statistically significant.

The overall heterogeneity was significantly high (I^2^ = 99%, p < 0.00001). The reason for such high heterogeneity might be the pooling of studies with different organisms; however, because we did not want to stratify our results based on the species rather than on the class of organism, this high heterogeneity did not affect our analysis.

Sensitivity Analysis

A sensitivity analysis was performed to determine the influence of each study on the overall effect. We excluded one study at a time, followed by the generation of pooled prevalence for the remaining studies. There was no significant effect of any study on the indicated robust results.

Discussion

In this systematic review and meta-analysis, we determined the prevalence of opportunistic infections in COVID-19 patients. The analysis showed the highest prevalence of viruses in COVID-19 patients, followed by bacteria and fungi. While screening articles for this study, interesting data were revealed by various studies on secondary infections and superinfections in COVID-19 patients.

In a study by Paolucci et al., a correlation between EBV load and COVID-19 severity was observed [27]. Out of 104 patients infected with SARS-CoV-2, 42 (40.4%) were hospitalized in an intensive care unit (ICU) and 62 (59.6%) in a sub-intensive care unit (SICU). Reactivation of human cytomegalovirus (HCMV) and EBV, parvovirus B19, and human herpesvirus 6 virus were determined by real-time polymerase chain reaction, whereas lymphocyte subpopulation counts were determined by flow cytometry. Among opportunistic viruses, only EBV was constantly identified. EBV DNA was determined in 40/42 (95.2%) of the ICU patients and in 51/61 (83.6%) of the SICU patients.

In another observation cohort study by Garcia-Vidal et al., few patients developed superinfections during hospitalization [15]. Out of the 989 patients with COVID-19, 72 (7.2%) had 88 other identified infections, of which 74 were bacterial, seven fungal, and seven viral. Community-acquired coinfection at the time of COVID-19 diagnosis was not common (31/989, 3.1%), and the primary pathogens were *Streptococcus pneumoniae* and *Staphylococcus aureus*. Overall, 51 hospital-acquired bacterial superinfections, mostly caused by *Pseudomonas aeruginosa* and *Escherichia coli*, were reported in 43 (4.7%) patients, with a mean (SD) time from hospital admission to superinfection diagnosis of 10.6 (6.6) days. These findings were different compared to those of other viral pandemics.

Søgaard et al. found that community-acquired viral and bacterial infections were uncommon among COVID-19 patients [18]. The outcome of ICU patients was complicated by hospital-acquired bacterial or fungal infections. In their cohort of 162 hospitalized patients with a median age of 64.4 years (interquartile range (IQR) = 50.4-74.2), 61.1% being male, 41 (25.3%) patients were admitted to the ICU, with 34/41 (82.9%) requiring mechanical ventilation, and 17 (10.5%) of all hospitalized patients died. A total of 31 infections were identified that comprised five viral coinfections, 24 bacterial infections, and three fungal infections (five ventilator-associated pneumonia, 13 tracheobronchitis, one pneumonia, and six bloodstream infections).

Sharifipour et al. emphasized superinfections in COVID-19 patients caused by *Acinetobacter baumannii* and *Staphylococcus aureus* [12]. In their study of 19 COVID-19 patients, all patients had bacterial infections, with 17 due to *Acinetobacter baumannii* (90%) and two due to *Staphylococcus aureus* (10%). None of the 17 strains with *Acinetobacter baumannii* infections were sensitive to the evaluated antibiotics.

Sharov studied two datasets [13]. Set 1 included the results of 3,382 assays of outpatients and hospital patients with community-acquired and hospital-acquired pneumonia of unknown etiology. Set 2 included the results of 1,204 assays of hospital patients with pneumonia and COVID-19-confirmed patients. Set 1 revealed 4.35% of all pneumonia cases were caused by SARS-CoV-2 with large mortality (18.75%) due to COVID-19. However, Set 2 showed that 52.82% of patients had other typical and atypical pathogens causing pneumonia. In total, 433 (35.96%) COVID-19 patients reported the presence of other bacteria, with *Streptococcus pneumoniae*, *Staphylococcus aureus*, and *Hemophilus influenzae* infections being the cause of secondary pneumonia.

Another cohort study by Ripa et al. described the incidence and predictive factors of secondary infections in patients with COVID-19 [16]. In their study, out of 731 patients, 68 (9.3%) patients had a secondary infection; at least one bloodstream infection was identified in 58/731 (7.9%) patients, and 22/731 (3.0%) patients had at least one possible lower respiratory tract infection (pLRTI). Secondary infection was frequently seen in patients admitted to ICU in two days (45/86) compared to those never admitted to the ICU or admitted to the ICU after two days. The incidence rate of BSIs was higher, that is, 31.9 (23.0-43.1) per 1,000 person-days of follow-ups (PDFUs) in patients admitted to the ICU compared to 3.3 (2.3-4.6) per 1,000 PDFUs in patients outside the ICU (p < 0.0001). Gram-positive pathogens (76/106 isolates, 71.7%) caused most of the bloodstream infections, especially coagulase-negative staphylococci (53/76, 69.7%), while among Gram-negatives (23/106, 21.7%) *Acinetobacter baumannii* (7/23, 30.4%) and *Escherichia coli* (5/23, 21.7%) were the dominant pathogens. The incidence rate of pLRTIs was higher, that is, 15.2 (9.3-23.4) per 1,000 PDFUs in patients inside the ICU compared to 0.4 (0.1-1.1) in patients outside the ICU (p < 0.0001). pLRTIs were primarily due to Gram-negative pathogens (14/26, 53.8%). Invasive aspergillosis was identified in 11 patients. Of the 11 patients, 10 were in the ICU when putative aspergillosis was diagnosed. On multivariable analysis, the factors related to secondary infections were low baseline lymphocyte count (≤0.7 versus >0.7 per 10^9^/L, subdistribution hazard ratios (sdHRs) of 1.93, 95% CI of 1.11-3.35), baseline PaO_2_/FiO_2_ (per 100 points lower = sdHRs 1.56, 95% CI = 1.21-2.04), and ICU admission in the first 48 hours (sdHRs = 2.51, 95% CI = 1.04-6.05).

Russell et al. analyzed data from 48,902 patients admitted to the hospital between February 6 and June 8, 2020 [17]. Pathogen determination was done for 8,649 (17.7%) out of the 48,902 patients, of which 1,107 patients were investigated for clinically significant COVID-19-associated respiratory or bloodstream culture. Out of 1,080 infections, 762 (70.6%) were secondary that occurred at least two days post-hospitalization. The primary respiratory coinfection pathogens were *Staphylococcus aureus* and *Hemophilus influenzae* (reported ≤2 days post-admission), whereas the predominant secondary respiratory infections were *Enterobacteriaceae *and *Staphylococcus aureus*. *Escherichia coli* and *Staphylococcus aureus* predominantly caused bloodstream infections.

Razazi et al. in a retrospective study compared the incidence of ventilator-associated pneumonia (VAP) and invasive aspergillosis among individuals with COVID-19-associated acute respiratory distress syndrome (C-ARDS) and those with non-SARS-CoV-2 viral ARDS (NC-ARDS) [19]. They evaluated mechanically ventilated 90 C-ARDS and 82 NC-ARDS patients. There were substantially fewer bacterial coinfections in the C-ARDS than in the NC-ARDS group: 14 (16%) versus 38 (48%) (p < 0.01) at the time of ICU admission. On the contrary, more patients suffered at least one episode of VAP in the C-ARDS group compared to the NC-ARDS: 58 (64%) versus 36 (44%) (p = 0.007). The probability of VAP was high in the C-ARDS group post-mortality adjustment and removal of the ventilator (sub-hazard ratio = 1.72 (1.14-2.52), p < 0.01). The C-ARDS group had a higher incidence of multidrug-resistant bacteria (MDR)-related VAP than the NC-ARDS group: 21 (23%) versus 9 (11%) (p = 0.03). The C-ARDS group received more carbapenem compared to NC-ARDS: 48 (53%) versus 21 (26%) (p < 0.01).

White et al. in their prospective cohort study determined the incidence, risk factors, and impact of invasive fungal infections in adult COVID-19 patients with severe respiratory distress [20]. Out of 135 COVID-19-positive individuals (with a median age of 57 and a male-to-female ratio of 2·2/1), 26.7% (14.1% aspergillosis, 12.6% yeast infections) were reported. The mortality rate was 38%; 53% in patients with fungal diseases and 31% in patients without fungal diseases (p = 0.0387). Antifungal treatment declined the mortality rate (38.5% in patients receiving therapy versus 90% in patients not receiving therapy; p = 0.008). The tendency of aspergillosis was increased with corticosteroids (p = 0.007) and medical history of chronic respiratory disease (p = 0.05). A fungal disease was observed often in critically ill COVID-19 patients.

The retrospective study by Li et al. among 1,495 patients hospitalized with COVID-19 showed that SBI was seen in 102 (6.8%) and about 50% of patients (49.0%, 50/102) died during hospitalization [21]. Compared to severe patients, the possibility of SBIs was high among critical patients. In total, 136 (85.5%) strains of Gram-negative bacteria were identified out of the 159 strains of bacteria isolated from the SBIs. The most common bacteria among the SBIs were *Acinetobacter baumannii* (35.8%, 57/159), *Klebsiella pneumoniae* (30.8%, 49/159), and *Stenotrophomonas maltophilia* (6.3%, 10/159). Overall, 91.2% was the isolation rate of carbapenem-resistant *Acinetobacter baumannii* whereas 75.5% was that of *Klebsiella pneumoniae*. *Staphylococcus aureus* and coagulase-negative staphylococci were methicillin-resistant. No vancomycin resistance was detected. This revealed that the incidence of SBIs in COVID-19 patients was related to the severity of illness on admission. The most common Gram-negative pathogens were *Acinetobacter baumannii* and *Klebsiella pneumoniae*. Moreover, the resistance rates of the dominant isolated bacteria were mostly high.

Bayram et al. in a prospective observational study presented the various characteristics of rhino-orbital mucormycosis (ROM) coinfection in severe COVID-19 patients [22]. In total, 11 positive ROM coinfection cases in severe COVID-19 patients were identified, of which seven (63.6%) cases of orbital apex syndrome and four (36.4%) cases of orbital cellulitis were identified. Overall, 54.5% of patients had endophthalmitis, with two patients suffering from retinoschisis.

COVID-19-associated pulmonary aspergillosis was investigated by Lahmer et al., Vélez Pintado et al., Segrelles-Calvo et al., and Gouzien et al. [23-26].

Lahmer et al. assessed the incidence, risk factors, and outcome of invasive pulmonary aspergillosis (IPA) in critically ill COVID-19 patients [23]. In total, 32 critically ill COVID-19 patients were screened for 28 days using a standardized protocol for the development of COVID-19-associated invasive pulmonary aspergillosis (CAPA). Overall, 11/32 (34%) of critically ill patients with severe COVID-19 pneumonia developed CAPA at the median of four days post ICU admission compared to 8% in the control cohort. In the COVID-19 cohort, patients who developed CAPA had higher mean age, Acute Physiology and Chronic Health Evaluation (APACHE) II score, and ICU mortality compared to the group without CAPA (36% versus 9.5%; p < 0.001). ICU stay (21 versus 17 days; p = 0.340) and days of mechanical ventilation (20 versus 15 days; p = 0.570) were similar among both groups. In regression analysis, COVID-19 and APACHE II scores were independently associated with IPA.

Vélez Pintado et al. performed a retrospective cohort study at a tertiary care center in Mexico City and reported that the CAPA was primarily seen in critically ill COVID-19 patients and was also related to an increased mortality rate [24]. Of the 83 ICU COVID-19 hospitalized patients, 16 (19.3%) met the criteria for CAPA. All CAPA individuals needed invasive mechanical ventilation (IMV) whereas only 84% of patients in the non-IPA group required IMV (p = 0.09). Mortality was reported in 31% (n = 5) of the IPA group whereas it was 13% (n = 9) in the non-CAPA group (p = 0.08).

Poor outcome was seen with CAPA in an open prospective observational study by Segrelles-Calvo et al. [25]. Of the total number of patients included in the study (n = 215), the authors diagnosed opportunistic invasive fungal infection in 49 (22.8%) patients. Seven of the patients had an infection caused by *Aspergillus* spp. (*Aspergillus fumigatus*, n = 3; *Aspergillus flavus*, n = 2 and *Aspergillus niger*, n = 20). The global prevalence of aspergillosis was 5.4%. Another retrospective cohort study by Gouzien et al. showed a significantly lower incidence of IPA (1.8%; 1/53) [26].

Based on the literature evidence, superinfections and respiratory coinfections in SARS-CoV-2-positive patients were more prevalent in critically ill COVID-19 patients. Among patient characteristics, a significant relationship was found in men with COVID-19. In their study, Paolucci et al. supported the correlation between lymphopenia and increased viral load (especially EBV), which shows the relationship between immunosuppression and viral prevalence [27]. Several studies [17,19,20] have reported increasing bacterial superinfections which could be due to antibiotic resistance, further warranting optimum ways to monitor antibiotic usage.

Limitations

Our study has certain limitations. COVID-19 is a novel disease, and its relationship between pathophysiology and patient presentation is not well understood. We examined COVID-19 patients with superinfections or opportunistic infections, and the occurrence of these infections in COVID-19 patients may not be correctly identified as there were no consistent screening tools used to identify these infections.

## Conclusions

Opportunistic infections are more prevalent in critically ill patients. The isolated pathogens included EBV, *Pseudomonas aeruginosa*, *Escherichia coli*, *Acinetobacter baumannii*, *Hemophilus influenzae*, and invasive pulmonary aspergillosis. Large-scale studies are required to estimate opportunistic/secondary/superinfections in COVID-19 patients.

## References

[1] (2022). Modes of transmission of virus causing COVID-19: implications for IPC precaution recommendations. https://www.who.int/news-room/commentaries/detail/modes-of-transmission-of-virus-causing-covid-19-implications-for-ipc-precaution-recommendations.

[2] Wang Y, Wang Y, Chen Y, Qin Q (2020). Unique epidemiological and clinical features of the emerging 2019 novel coronavirus pneumonia (COVID-19) implicate special control measures. J Med Virol.

[3] Chertow DS, Memoli MJ (2013). Bacterial coinfection in influenza: a grand rounds review. JAMA.

[4] Morens DM, Taubenberger JK, Fauci AS (2008). Predominant role of bacterial pneumonia as a cause of death in pandemic influenza: implications for pandemic influenza preparedness. J Infect Dis.

[5] Lin D, Liu L, Zhang M (2020). Co-infections of SARS-CoV-2 with multiple common respiratory pathogens in infected patients. Sci China Life Sci.

[6] Nowak MD, Sordillo EM, Gitman MR, Paniz Mondolfi AE (2020). Coinfection in SARS-CoV-2 infected patients: where are influenza virus and rhinovirus/enterovirus?. J Med Virol.

[7] Wang M, Wu Q, Xu W (2020). Clinical diagnosis of 8274 samples with 2019-novel coronavirus in Wuhan. medRxiv.

[8] Fishman JA (2013). Opportunistic infections--coming to the limits of immunosuppression?. Cold Spring Harb Perspect Med.

[9] Musuuza JS, Watson L, Parmasad V, Putman-Buehler N, Christensen L, Safdar N (2021). Prevalence and outcomes of co-infection and superinfection with SARS-CoV-2 and other pathogens: a systematic review and meta-analysis. PLoS One.

[10] Hutton B, Salanti G, Caldwell DM (2015). The PRISMA extension statement for reporting of systematic reviews incorporating network meta-analyses of health care interventions: checklist and explanations. Ann Intern Med.

[11] Higgins JP, Thompson SG, Deeks JJ, Altman DG (2003). Measuring inconsistency in meta-analyses. BMJ.

[12] Sharifipour E, Shams S, Esmkhani M (2020). Evaluation of bacterial co-infections of the respiratory tract in COVID-19 patients admitted to ICU. BMC Infect Dis.

[13] Sharov KS (2020). SARS-CoV-2-related pneumonia cases in pneumonia picture in Russia in March-May 2020: secondary bacterial pneumonia and viral co-infections. J Glob Health.

[14] Asmarawati TP, Rosyid AN, Suryantoro SD (2021). The clinical impact of bacterial co-infection among moderate, severe and critically ill COVID-19 patients in the second referral hospital in Surabaya. F1000Res.

[15] Garcia-Vidal C, Sanjuan G, Moreno-García E (2021). Incidence of co-infections and superinfections in hospitalized patients with COVID-19: a retrospective cohort study. Clin Microbiol Infect.

[16] Ripa M, Galli L, Poli A (2021). Secondary infections in patients hospitalized with COVID-19: incidence and predictive factors. Clin Microbiol Infect.

[17] Russell CD, Fairfield CJ, Drake TM (2021). Co-infections, secondary infections, and antimicrobial use in patients hospitalised with COVID-19 during the first pandemic wave from the ISARIC WHO CCP-UK study: a multicentre, prospective cohort study. Lancet Microbe.

[18] Søgaard KK, Baettig V, Osthoff M (2021). Community-acquired and hospital-acquired respiratory tract infection and bloodstream infection in patients hospitalized with COVID-19 pneumonia. J Intensive Care.

[19] Razazi K, Arrestier R, Haudebourg AF (2020). Risks of ventilator-associated pneumonia and invasive pulmonary aspergillosis in patients with viral acute respiratory distress syndrome related or not to Coronavirus 19 disease. Crit Care.

[20] White PL, Dhillon R, Cordey A (2021). A national strategy to diagnose coronavirus disease 2019-associated invasive fungal disease in the intensive care unit. Clin Infect Dis.

[21] Li J, Wang J, Yang Y, Cai P, Cao J, Cai X, Zhang Y (2020). Etiology and antimicrobial resistance of secondary bacterial infections in patients hospitalized with COVID-19 in Wuhan, China: a retrospective analysis. Antimicrob Resist Infect Control.

[22] Bayram N, Ozsaygılı C, Sav H (2021). Susceptibility of severe COVID-19 patients to rhino-orbital mucormycosis fungal infection in different clinical manifestations. Jpn J Ophthalmol.

[23] Lahmer T, Kriescher S, Herner A (2021). Invasive pulmonary aspergillosis in critically ill patients with severe COVID-19 pneumonia: results from the prospective AspCOVID-19 study. PLoS One.

[24] Vélez Pintado M, Camiro-Zúñiga A, Aguilar Soto M, Cuenca D, Mercado M, Crabtree-Ramirez B (2021). COVID-19-associated invasive pulmonary aspergillosis in a tertiary care center in Mexico City. Med Mycol.

[25] Segrelles-Calvo G, Araújo GR, Llopis-Pastor E (2021). Prevalence of opportunistic invasive aspergillosis in COVID-19 patients with severe pneumonia. Mycoses.

[26] Gouzien L, Cocherie T, Eloy O (2021). Invasive Aspergillosis associated with Covid-19: a word of caution. Infect Dis Now.

[27] Paolucci S, Cassaniti I, Novazzi F (2021). EBV DNA increase in COVID-19 patients with impaired lymphocyte subpopulation count. Int J Infect Dis.

[28] Zhang H, Zhang Y, Wu J (2020). Risks and features of secondary infections in severe and critical ill COVID-19 patients. Emerg Microbes Infect.

[29] Bardi T, Pintado V, Gomez-Rojo M (2021). Nosocomial infections associated to COVID-19 in the intensive care unit: clinical characteristics and outcome. Eur J Clin Microbiol Infect Dis.

[30] Falcone M, Tiseo G, Giordano C (2021). Predictors of hospital-acquired bacterial and fungal superinfections in COVID-19: a prospective observational study. J Antimicrob Chemother.

[31] Khurana S, Singh P, Sharad N (2021). Profile of co-infections & secondary infections in COVID-19 patients at a dedicated COVID-19 facility of a tertiary care Indian hospital: Implication on antimicrobial resistance. Indian J Med Microbiol.

[32] Kubin CJ, McConville TH, Dietz D (2021). Characterization of bacterial and fungal infections in hospitalized patients with coronavirus disease 2019 and factors associated with health care-associated infections. Open Forum Infect Dis.

[33] Kumar G, Adams A, Hererra M (2021). Predictors and outcomes of healthcare-associated infections in COVID-19 patients. Int J Infect Dis.

